# The Role of Video-Assisted Thoracoscopic Therapeutic Resection for Medically Failed Pulmonary Tuberculosis

**DOI:** 10.1097/MD.0000000000003511

**Published:** 2016-05-06

**Authors:** Yau-Lin Tseng, Jia-Ming Chang, Yi-Sheng Liu, Lili Cheng, Ying-Yuan Chen, Ming-Ho Wu, Chung-Lan Lu, Yi-Ting Yen

**Affiliations:** From the Department of Surgery, Division of Thoracic Surgery, National Cheng Kung University Hospital, College of Medical College, National Cheng Kung University, Tainan (Y-LT, Y-YC, Y-TY); Department of Surgery, Division of Thoracic Surgery, Chia-Yi Christian Hospital, Chia-Yi (J-MC); Graduate Institute of Medical Sciences, Collage of Health Science, Chang Jung Christian University, Tainan (J-MC); Department of Diagnostic Radiology, National Cheng Kung University Hospital, College of Medical College, National Cheng Kung University (Y-SL, LC); Department of Surgery, Division of Thoracic Surgery, Tainan Municipal Hospital (M-HW); Center for Infection Control, National Cheng Kung University Hospital (C-LL); and Institute of Clinical Medicine, College of Medical College (J-MC, Y-TY), National Cheng Kung University, Tainan, Taiwan.

## Abstract

There are few reports regarding video-assisted thoracoscopic therapeutic resection for medically failed pulmonary tuberculosis (TB). We reviewed our surgical results of video-assisted thoracoscopic surgery (VATS) therapeutic resection for pulmonary TB with medical failure, and its correlation with image characteristics on chest computed tomography (CT) scan.

Between January 2007 and December 2012, among the 203 patients who had surgery for TB, the medical records of 89 patients undergoing therapeutic resection for medically failed pulmonary TB were reviewed. Clinical information and the image characteristics of CT scan were investigated and analyzed.

Forty-six of the 89 patients undergoing successful VATS therapeutic resection had significantly lower grading in pleural thickening (*P* < 0.001), peribronchial lymph node calcification (*P* < 0.001), tuberculoma (*P* = 0.015), cavity (*P* = 0.006), and aspergilloma (*P* = 0.038); they had less operative blood loss (171.0 ± 218.7 vs 542.8 ± 622.8 mL; *P* < 0.001) and shorter hospital stay (5.2 ± 2.2 vs 15.6 ± 15.6 days; *P* < 0.001). They also had a lower percentage of anatomic resection (73.9% vs 93.0%; *P* = 0.016), a higher percentage of sublobar resection (56.5% vs 32.6%; *P* = 0.023), and a lower disease relapse rate (4.3% vs 23.3%; *P* = 0.009). Eighteen of the 38 patients with multi-drug resistant pulmonary tuberculosis (MDRTB) who successfully underwent VATS had significantly lower grading in pleural thickening (*P* = 0.001), peribronchial lymph node calcification (*P* = 0.019), and cavity (*P* = 0.017). They were preoperatively medicated for a shorter period of time (221.6 ± 90.8 vs 596.1 ± 432.5 days; *P* = 0.001), and had more sublobar resection (44.4% vs 10%), less blood loss (165.3 ± 148.3 vs 468.0 ± 439.9 mL; *P* = 0.009), and shorter hospital stay (5.4 ± 2.6 vs 11.8 ± 6.9 days; *P* = 0.001).

Without multiple cavities, peribronchial lymph node calcification, and extensive pleural thickening, VATS therapeutic resection could be safely performed in selected patients with medically failed pulmonary TB as an effective adjunct with satisfactory results.

## INTRODUCTION

Despite the improvement of medication, surgical intervention has been advocated as an effective adjunct in the treatment for pulmonary tuberculosis (TB) and its complications.^1–3^ Since its introduction in the 1980 s, video-assisted thoracoscopic surgery (VATS) has evolved and become the preferred approach in thoracic surgery over the past decade. In fact, VATS has been documented as both diagnostically and therapeutically useful and feasible for selected patients with pulmonary TB.^4,5^ It is an existing preconception that pleural complications of pulmonary TB are more likely to be managed with VATS than parenchymal destruction deemed for surgical resection. The application of video-assisted thoracoscopic therapeutic resection has not been elucidated in the treatment of medically failed pulmonary TB.

Our university hospital is a referral center for complicated pulmonary TB. Since January 2007, VATS has been our preferred method for thoracic surgery, not only for patients with malignancies, but also for those with complications of pulmonary TB. Notably, VATS therapeutic resections were then reimbursed by the National Health Insurance Administration, and therefore all the therapeutic resections for pulmonary TB were initially attempted with the thoracoscopic approach. Conversion to thoracotomy was done if thoracoscopic adhesiolysis or a hilar dissection was intraoperatively evaluated as difficult. We previously reported that image characteristics of computed tomography (CT) predicted the feasibility of VATS anatomic lung resection in patients with pulmonary TB, and VATS anatomic lung resection could be done in selected patients with satisfactory results.^6,7^ We herein retrospectively review the applicability and surgical results of VATS in the treatment of patients with medically failed TB, and delineate the association with image characteristics of chest CT scan.

## MATERIALS AND METHODS

### Patient Enrollment

Between January 2007 and December 2012, among the 203 patients who had surgery for pulmonary TB, the medical records of 89 patients who underwent therapeutic resection for medically failed pulmonary TB were retrospectively reviewed. Those who underwent concomitant decortication or thoracoplasty, bronchoscopy for blood clot evacuation, or lung resections for complications without active infection were excluded. Patients with medically failed pulmonary TB included 3 groups: those who were sputum smear- or sputum culture-positive at 4 months or later after the initiation of anti-TB treatment, those with drug-resistant strain and radiographic cavitation, and those with unresolved radiographic lesion despite of culture-guided medication with disease relapse or high risk of disease relapse. Patients of high-risk treatment failure or disease relapse were those with pulmonary TB, diabetes mellitus, and a cavitary lesion in the lung on the image study. Patients were also considered of high risk if they had pulmonary TB of localized extent and diabetes mellitus but not readily accessible to the appropriate medications, which needed to be specifically imported, such as kanamycin, streptomycin, capreomycin, terizidone, and clofazimine. Panel discussions between board-certified chest physicians specialized in pulmonary TB and thoracic surgeons under the surveillance of Center for Disease Control, Ministry of Health and Welfare, were routinely held and collaborated with Center for Infection Control in National Cheng Kung University Hospital. Sputum acid-fast stain and culture were routinely obtained after the patients had been admitted for surgery and before surgery. Informed consent was waived because the study was retrospective, and the review of medical records was approved by the Institutional Review Board of National Cheng Kung University Hospital (B-ER-101–080). Clinical information was collected on age, sex, the ultimate surgical approach and procedures, operative time, blood loss, length of hospital stay, complication, treatment outcome, and follow-up duration. Preoperative sputum yields were also investigated and recorded. Patients newly diagnosed with pulmonary TB, or who had been operated on because of treatment failure, were given anti-TB medications for 6 additional months; those who had been operated on because of multi-drug resistant TB (MDRTB) were given anti-TB medications for at least 24 months after the sputum culture had become negative for TB. The regimen of anti-TB medications was directed according to the Taiwan Guideline for TB Diagnosis and Treatment.^8^

### Classification and Grading of Image Characteristics

The preoperative chest CT scans were reviewed by 2 board-certified radiologists specialized in thoracic images, who were blinded to the patients’ identities and surgical procedures. Abnormal findings identified on the chest CT scans were interpreted and classified as bulla, pleural thickening, peribronchial lymph node calcification, tuberculoma, cavity, aspergilloma, atelectasis, and bronchiectasis, with the radiographic criteria as reported in Table 1 and our previous article.^6^ Pleural thickness was measured from the mediastinal window images. All the image characteristics were graded according to the number of lesions and degree of lobar involvement.

**TABLE 1:**
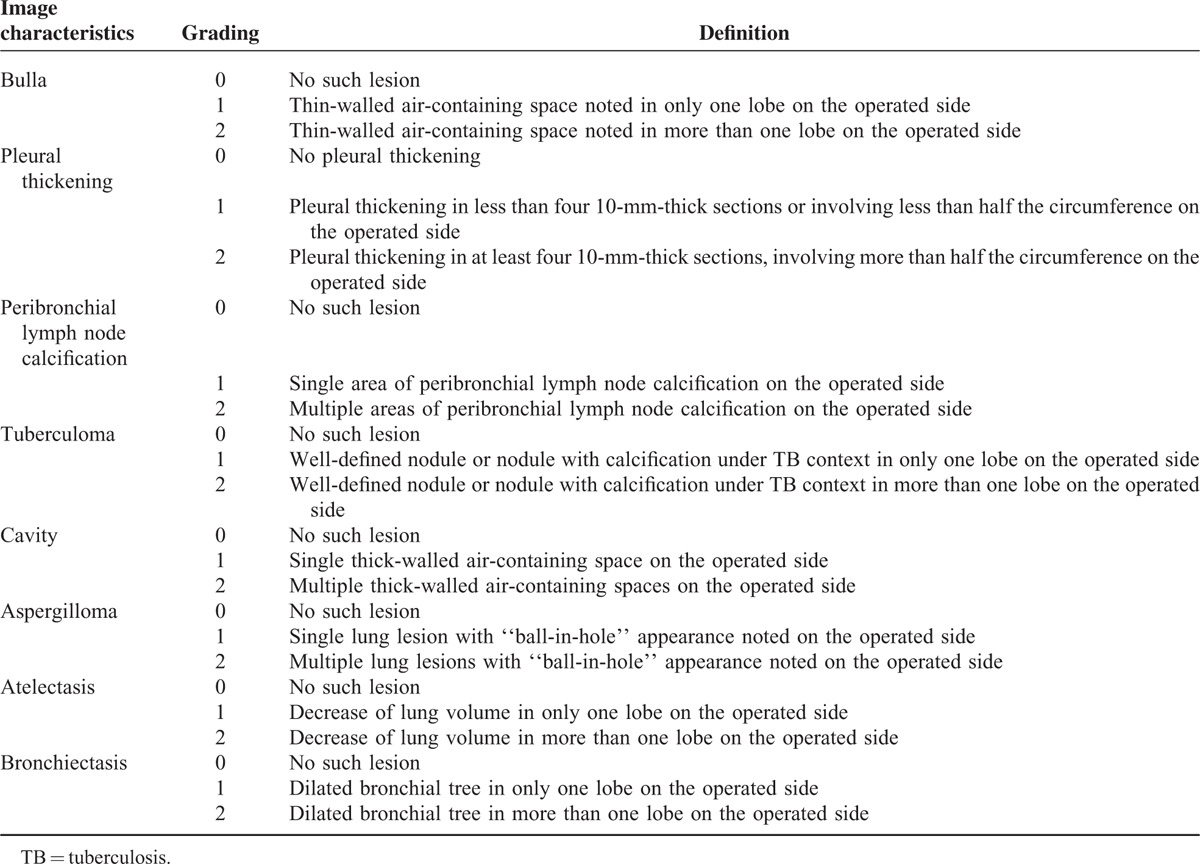
Definition and Grading of Image Characteristics on Chest Computed Tomographic Scan in Patients Who Underwent Anatomical Lung Resection for TB

### Techniques

All the surgeries were done by Y-LT and M-HW, who were both experienced in TB lung surgery,^9^ and assisted by J-MC, Y-YC, or Y-TY. An anatomic resection is defined as a procedure requiring dissection and ligation of vascular and bronchial structure, including pneumonectomy, lobectomy, and segmentectomy. A sublobar resection is defined as the extent of resection less than a lobe, including segmentectomy and wedge resection. The patient was placed in the full lateral decubitus position under general anesthesia with selective one-lung ventilation. All the surgeries started with VATS, but converted to thoracotomy, including posteriolateral or anterior thoracotomy, if the dense adhesion in the pleural space or around hilar structures precluded VATS. A thoracostomy at the 6th intercostal space in the mid-axillary line was created for the flexible (EVE-L; Fujinon, Wayne, NJ) or rigid (Karl Storz Endoscopy-America, Culver City, CA) 30-degree thoracoscope through a 10.5-mm trocar. A utility thoracotomy of 3 to 5 cm at the third or fourth intercostal space was created and protected with a wound retractor (Alexis; Applied Medical, Rancho Santa Margarita, CA). Another thoracostomy was placed in varying locations for traction and countertraction. A scalpel and electrocautery (Harmonic ACE^®^; Johnson & Johnson, Somerville, NJ) were used for pleural adhesiolysis. The major vascular branches were transected with a stapler (Echelon Flex 45 Endopath; Johnson & Johnson, New Brunswick, NJ) and staples (ECR45W white reloads; Johnson & Johnson), whereas minor branches were ligated with silk, a knot pusher, and clips. The lobar bronchus was closed with staples (TA 30 [4.8]; Johnson & Johnson) and reinforced with 4–0 sutures (Maxon; Johnson & Johnson) in the thoracotomy group. In the VATS group, the same stapler was used to transect the lobar bronchus with ECR45G green reloads, and the segmental bronchus with ECR45D gold reloads.

### Statistics

Patients were divided into 2 groups: those who were successfully treated with VATS and those who were converted to thoracotomy because of difficult pleural adhesiolysis or hilar dissection. We also specifically evaluated the feasibility of VATS therapeutic resection in patients with MDRTB. Continuous variables between these 2 groups (age, percentage of preoperative positive sputum culture, operative time, blood loss, length of hospital stay, duration of preoperative medication, and postoperative follow-up) were compared using Student *t* test, whereas noncontinuous variables (sex, procedures, complication, and treatment result) were compared using a *χ*^2^ test. Statistical significance was set at *P* < 0.05.

## RESULTS

Forty-six of the 89 patients with medically failed TB successfully underwent therapeutic resection with VATS and the rest 43 (48.3%) were converted to thoracotomy. The distribution of sex and age was not significantly different as listed in Table 2. The thoracotomy group had a significantly higher percentage of anatomic resection than the VATS group (93.0% vs 73.9%; *P* = 0.016), whereas the VATS group had a significantly higher percentage of sublobar resection than the thoracotomy group (56.5% vs 32.6%; *P* = 0.023; Table 2). Patients undergoing pneumonectomy were all performed by thoracotomy instead of VATS (*P* = 0.034). However, significantly more wedge resections were performed in the VATS group than those in the thoracotomy group (20 vs 9; *P* = 0.01). Twenty-two of the 46 patients in the VATS group and 28 of the 43 in the thoracotomy group were preoperatively positive for sputum culture after they had been admitted for surgery, the proportion of which was not significantly different (47.8% vs 65.1%; *P* = 0.124). Although the operative time was not significantly different between the VATS and thoracotomy group, the VATS group had less operative blood loss (171.0 ± 218.7 vs 542.8 ± 622.8 mL; *P* < 0.001) and shorter hospital stay (5.2 ± 2.2 vs 15.6 ± 15.6 days; *P* < 0.001).The postoperative follow-up duration of the VATS group, however, was significantly shorter (1101 ± 586 vs 2136 ± 440 days; *P* < 0.001) than the thoracotomy group. The disease relapse rate was significantly lower in the VATS group than in the thoracotomy group (4.3% vs 23.3%; *P* = 0.009).

**TABLE 2:**
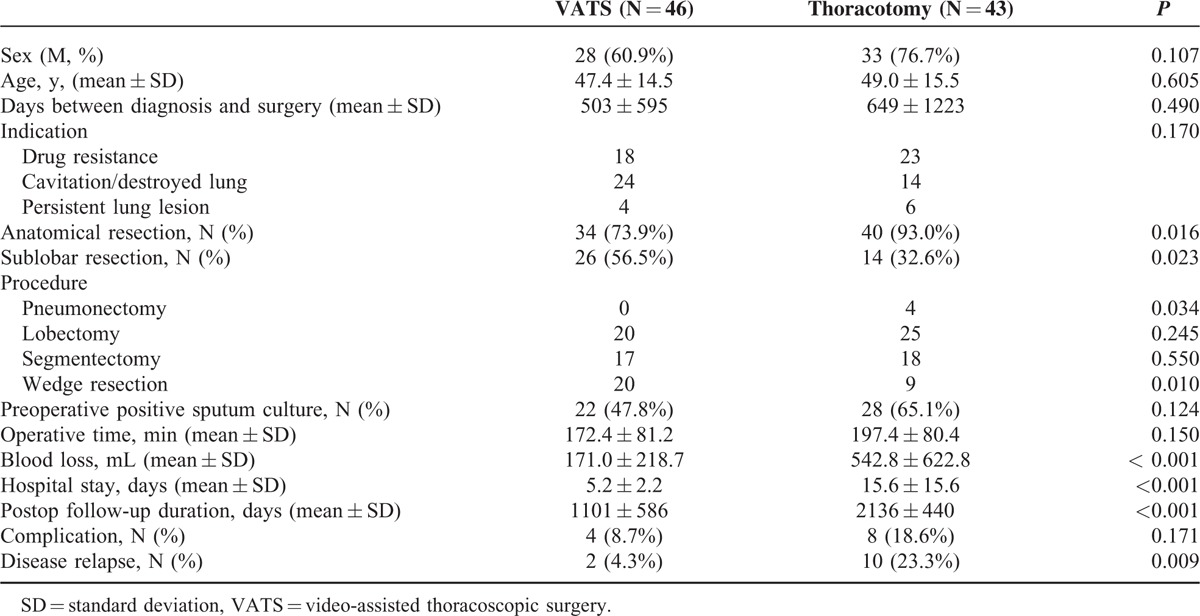
Characteristics of Patients With Pulmonary Tuberculosis Undergoing Therapeutic Resection for Medical Treatment Failure

Patients with MDRTB undergoing therapeutic resection were further analyzed as listed in Table 3. Eighteen patients with MDRTB underwent VATS therapeutic resection successfully, whereas 20 (52.6%) were converted to thoracotomy. The distribution of sex and age was not significantly different. The VATS group had a significantly shorter duration of anti-TB medication before surgery than the thoracotomy group (221.6 ± 90.8 vs 596.1 ± 432.5 days; *P* = 0.001; Table 3). Sixteen of the 18 patients in the VATS group and all of the 20 patients in the thoracotomy group underwent anatomic resection. Eight of the 18 patients in the VATS group and 2 of the 20 patients in the thoracotomy group underwent sublobar resection, and the percentage was significantly higher in the VATS group (44.4% vs 10%; *P* = 0.016). Pneumonectomy was performed in 2 patients with thoracotomy but not with VATS. Ten of the 18 patients in the VATS group and 13 of the 20 in the thoracotomy group were preoperatively positive for sputum culture after they had been admitted for surgery, the proportion of which was not significantly different (55.6% vs 65%; *P* = 0.552). Although the operative time was not significantly different between the VATS and thoracotomy group, the VATS group had less operative blood loss (165.3 ± 148.3 vs 468.0 ± 439.9 mL; *P* = 0.009) and shorter hospital stay (5.4 ± 2.6 vs 11.8 ± 6.9 days; *P* = 0.001). The VATS group, however, had a significantly shorter postoperative follow-up duration (1431 ± 478 vs 2414 ± 393 days; *P* < 0.001) than the thoracotomy group.

**TABLE 3:**
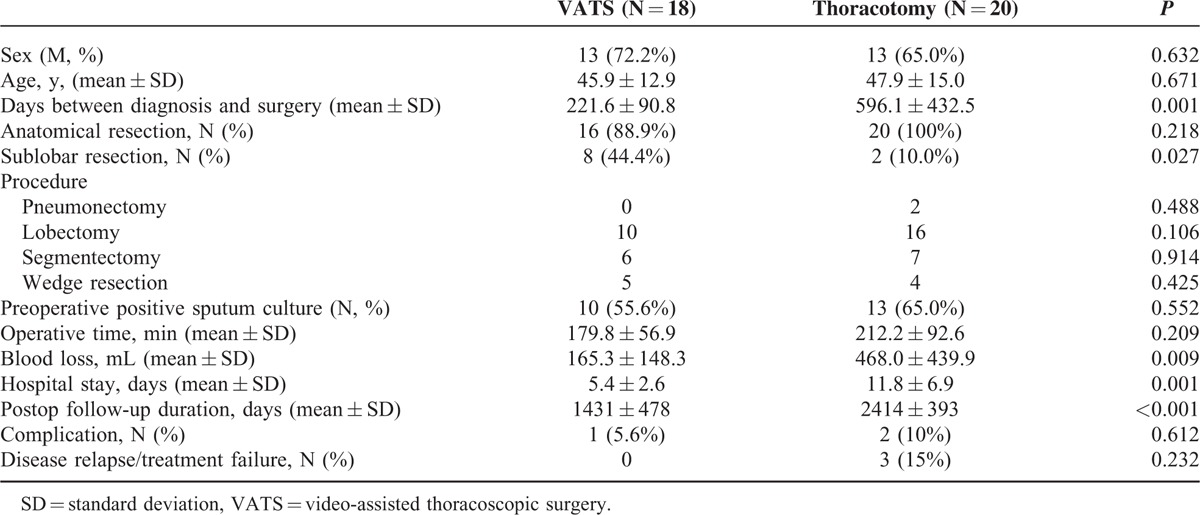
Characteristics of Patients With Multidrug-Resistant Pulmonary Tuberculosis Undergoing Therapeutic Resection

The image characteristics were further analyzed. Patients who successfully underwent VATS therapeutic resection had lower grading of pleural thickening (*P* < 0.001), peribronchial lymph node calcification (*P* < 0.001), tuberculoma (*P* = 0.015), cavity (*P* = 0.006), and aspergilloma (*P* = 0.038) than those converted to thoracotomy. (Table 4) The grading of other image characteristics such as bullae, atelectasis, and bronchiectasis was not significantly different between the VATS and thoracotomy groups (*P* = 0.239, 0.262, and 0.980, respectively; Table 4). Moreover, patients with MDRTB undergoing successful VATS therapeutic resection demonstrated lower grading in pleural thickening, peribronchial lymph node calcification, and cavity (*P* = 0.001, 0.019, 0.017, respectively; Table 5) than those converted to thoracotomy. The grading of other image characteristics such as bullae, tuberculoma, aspergilloma, atelectasis, and bronchiectasis was not significantly different between the VATS and thoracotomy groups in patients with MDRTB (*P* = 0.425, 0.155, 0.198, 0.485, and 0.544, respectively; Table 5).

**TABLE 4:**
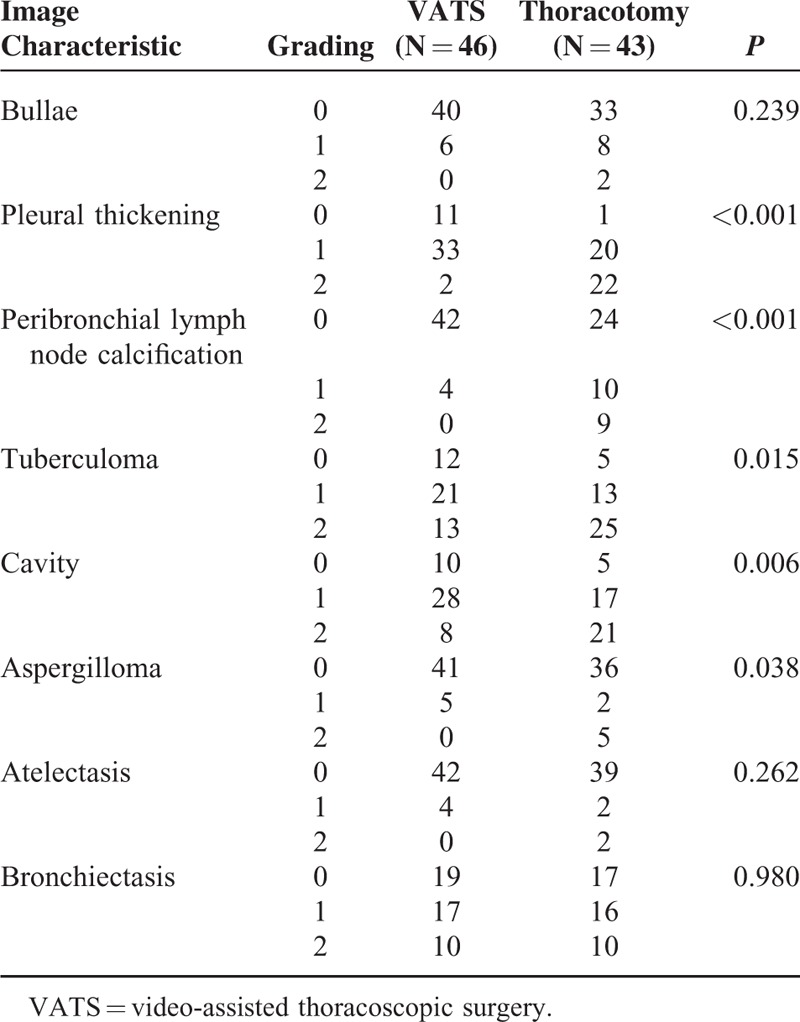
Image Characteristics of Patients With Pulmonary Tuberculosis Undergoing Therapeutic Resection for Medical Treatment Failure

**TABLE 5:**
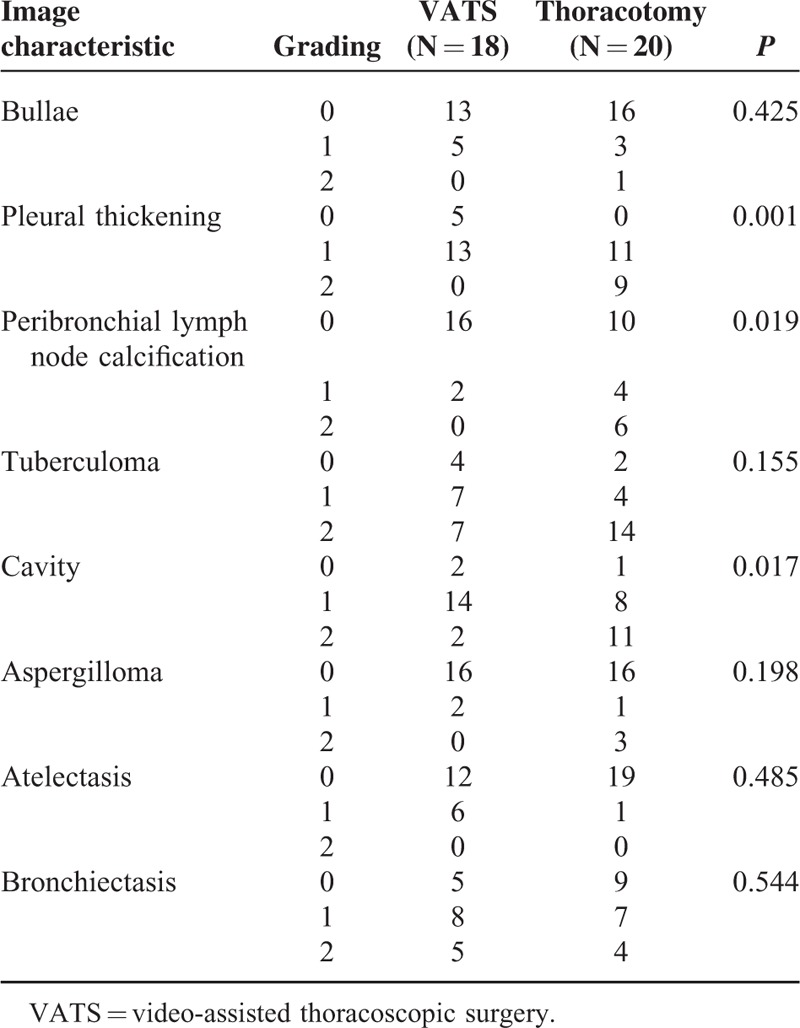
Image Characteristics of Patients With Multidrug-Resistant Pulmonary Tuberculosis Undergoing Therapeutic Resection

There were 4 surgical complications in the VATS group and 8 in the thoracotomy group (Table 6). One in the VATS group and 2 in the thoracotomy group had MDRTB. While 1 patient with complication in the VATS group was preoperatively positive for sputum culture after he had been admitted for surgery, so were 5 of the 8 patients in the thoracotomy group. Complications in the VATS group included hemothorax and bleeding from the bronchial artery, which occurred postoperatively and were controlled with the thoracoscopic approach. One patient had wound infection and disruption, which was managed with wound debridement and re-approximation. Left vocal cord palsy was identified in another patient, which was managed with intracordal injection. One patient in the thoracotomy group had excessive bleeding during pneumonectomy, which resulted in multiple organ failure and death. Multiple organ failure developed in another patient secondary to massive hemoptysis, hypoxia, and the resultant multiple organ failure despite of timely and successful therapeutic resection. Two patients had prolonged air leak postoperatively, which were managed conservatively.

**TABLE 6:**
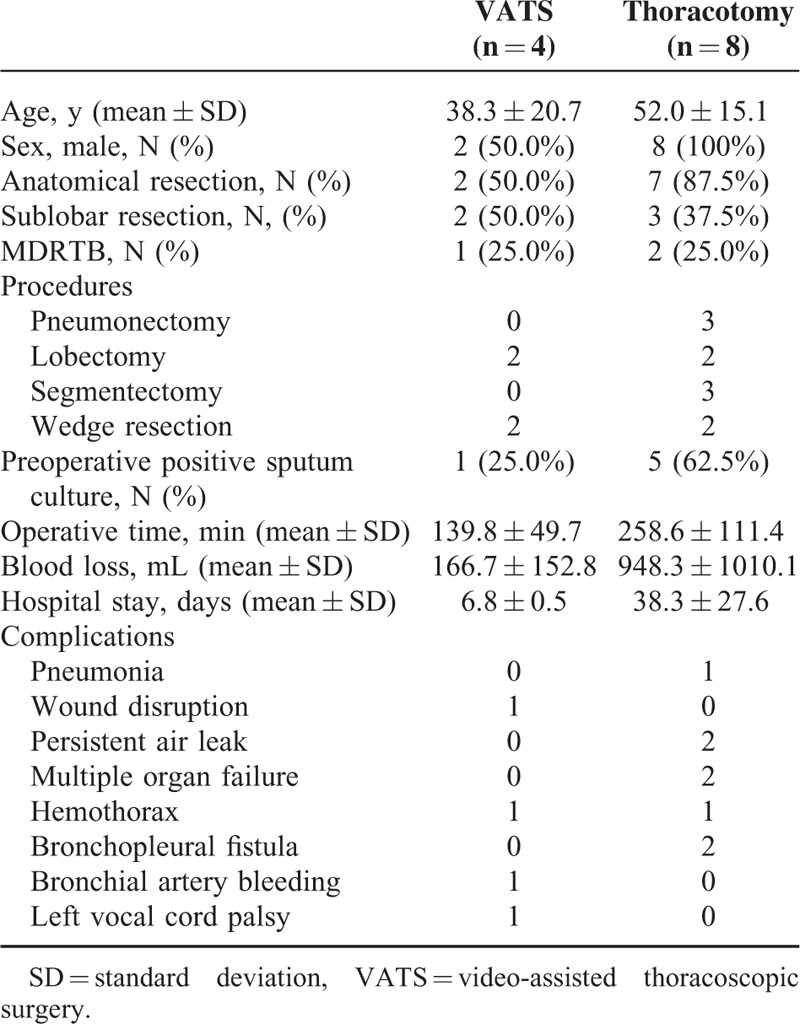
Surgical Complications of Patients Undergoing Therapeutic Resection for Pulmonary Tuberculosis With Medical Failure

Patients undergoing simple anatomic resection were compared with those undergoing combined anatomic resection, combined anatomic with nonanatomic resection, and simple nonanatomic resection (Table 7). Although 49 of all the 89 patients underwent simple anatomic resection, 23 of the 49 were successfully performed with VATS. Thoracotomy was done in 8 of the 11 patients undergoing combined anatomic resection, whereas VATS was successfully performed in 6 of the 14 patients undergoing combined anatomic and non-anatomic resection. Of note, VATS was performed in 12 of the 15 patients undergoing simple nonanatomic resection. The proportion of successful VATS resection was significantly different (*P* = 0.045). The grading of image characteristics was also calculated and analyzed, and only the grading of tuberculoma was significantly different among these 4 groups (*P* = 0.008).

**TABLE 7:**
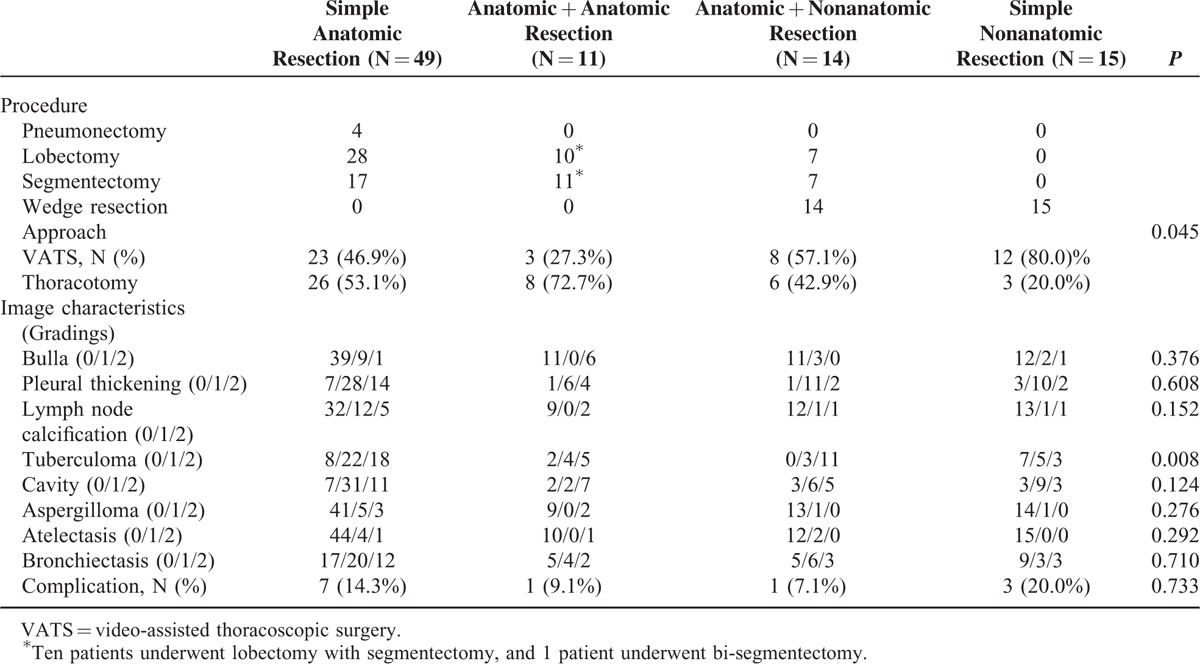
Comparison of Image Characteristics of Patients Undergoing Simple Anatomic Resection, Anatomic With Anatomic Resection, Anatomic With Nonanatomic Resection, and Simple Nonanatomic Resection

## DISCUSSION

Surgical resection has been advocated as an effective adjunct in the treatment of drug-resistant pulmonary TB^10,11^ and favorable outcomes of patients with MDRTB were reported to be up to 90%.^12^ Although a meta-analysis concluded that there was insufficient evidence to recommend surgery plus chemotherapy over chemotherapy alone, the results suggested that surgical intervention offered therapeutic benefit when the disease progressed beyond the reach of medical treatment.^13^ The rationale behind surgery is that resecting lesions harboring actively replicating bacilli dramatically reduces the overall organism burden in the lung while simultaneously removing the sites of compromised drug penetration. Thick-walled cavities and areas of destroyed lung contain up to 10^7^ to 10^9^ organisms even in patients who are sputum culture-negative.^14,15^ These lesions, because of their reduced exposure to host defense and anti-TB medications,^16^ are likely to provide an environment conducive to drug resistance.^17^ Surgical resection, by removing a localized burden of bacilli present in necrotic and nonviable lung tissue, enhances the efficacy of medical treatment, prevents further disease spread, and decreases the likelihood of drug resistance.

We demonstrated that even though around 50% of patients undergoing VATS therapeutic resection were converted to thoracotomy, patients with medically failed pulmonary TB could benefit from VATS before complicated and irreversible parenchymal damage occurred. Since 2007, when the video-assisted thoracoscopic therapeutic resection was reimbursed by the National Health Insurance Administration, VATS has been our preferred method for both patients with malignancies, and those with complications of pulmonary TB. The advantages of VATS, such as less wound pain, fewer pulmonary complications, and a shorter hospital stay than with a thoracotomy, have been strongly suggested.^18–21^ The reimbursement of VATS did encourage thoracic surgeons to further its application from malignant to infectious lung disease, conveying the benefit of minimally invasive surgery to patients with pulmonary TB, especially those who were often socioeconomically compromised and would have otherwise been deprived of the chance of cure because of the cost of surgery. The VATS therapeutic resection was initially attempted more on patients with pulmonary complications such as symptomatic cavitation or destroyed lung without active infection than on patients with medical failure. When we gradually became experienced, more liberal use of VATS was then conducted on patients with medically failed pulmonary TB. As patients had been properly selected and surgically treated, recovered uneventfully, and stayed back on the course of medical treatment, it would be persuasive for a multidisciplinary team of experts to reach a surgical decision whenever indicated. As early referral and surgical interventions became the routine, patients undergoing successful VATS therapeutic resection had not only lower grading of image characteristics, but also subsequently less blood loss and shorter hospital stay. With the evolution of surgical decision taken into account, it is understandable that the postoperative follow-up duration was significantly shorter in the VATS group. The disease relapse rate was also significantly lower in the VATS group of patients with medically failed pulmonary TB. Although the shorter postoperative follow-up duration might have confounded this result, compared with the thoracotomy group, early referral and liberal use of VATS might well pose substantial impact on decreasing the bacterial burden without much trauma, which enhanced the chance of cure.

The surgical indication for pulmonary TB with medical failure has remained largely unchanged since it was first described in 1990.^22^ Surgical candidates included patients with localized disease and adequate pulmonary reserve who had either persistently positive sputum smears or cultures despite appropriate medical treatment. The timing of surgery was considered to coincide with the lowest bacterial burden so that the outcome could be optimized with the least morbidity. Two to 3 months of effective medication and sputum culture conversion may lead to better outcome and a reduced risk of transmission to the operating room staff.^23^ However, surgery performed later in the course of disease might allow for longer treatment with anti-TB medication, nutritional supplementation, and control of coexisting medical illnesses, hence promising postoperative recovery. However, waiting to perform surgery and prolonging medical treatment may result in disease progression and even the emergence of drug resistance.^24^ Although sputum culture conversion within the first 2 to 3 months of treatment predicts better treatment outcome, conversion is not achievable without surgical intervention in some patients.^25,26^ Despite the fact that thoracoscopic approach might not be suitable for anatomic lung resection in patients with pulmonary TB,^4,5^ we demonstrated that VATS could be safely performed in meticulously selected patients by avoiding late-stage disease and irreversible morphologic changes in the lung parenchyma.^6,7^

As 80% of the simple wedge resections were performed using VATS, the comparison between the VATS and the thoracotomy group could be invalid because the latter had diseases of greater extent. The significantly smaller amount of operative blood loss and shorter hospital stay were likely the consequences of the less complex parenchymal status. Nonetheless, it would be practical for thoracic surgeons willing to perform VATS therapeutic resection for patients with medically failed pulmonary TB, to start with VATS wedge resection. Even though variable degrees of pleural adhesion encountered, the amount of blood loss and the risk of complications will significantly be decreased because of the facilitated dissection by the sharp vision of the VATS system. The information thoracic surgeons gained by performing VATS wedge resection for medically failed pulmonary TB will enhance their capabilities in dealing with the more technically demanding VATS anatomic lung resection. We previously reported that the presence of severe pleural adhesion and multiple cavities significantly increased the surgical risk during pulmonary resection for pulmonary TB.^27,28^ Some factors indeed hinder VATS anatomic resection for pulmonary TB. The presence of a chronically thickened cavity wall with obliterated pleural space in a destroyed lung should be treated with cavernostomy and thoracoplasty or cavernostomy with intrathoracic muscle flap transposition instead of VATS.^29^ In addition, extensive pleural adhesion (pleural thickening in at least four 10-mm-thick sections, involving more than half the circumstance on the operated side) and peribronchial lymph node calcification were highly indicative of conversion to a thoracotomy. Vessels firmly adhering to the bronchus or uncalcified lymph node, preoperatively unpredictable yet rarely encountered, may preclude VATS intraoperatively. Granulomatous involvement and the resultant calcification may cause the lymph nodes to adhere markedly to the vascular and bronchial structure, combined with distortion of the lobar anatomy. These consequences, necessitating tactile feedback for detailed dissection, make the dissection hazardous with the thoracoscopic approach and often fail VATS. Patients with a centrally located cavity or aspergilloma within one lobe, or with sputum-positive tuberculoma confined to one lobe, are good candidates for VATS anatomic resection. Larger tuberculoma within 1 lobe could also be resected using VATS, taking a larger incision to facilitate the dissection and extraction of the specimen. Based on the image characteristics, it is predictable that patients with severe pleural thickening, significant peribronchial lymph node calcification, or multiple cavities should be taken directly to thoracotomy instead of VATS. In other words, with the exclusion of the aforementioned lesions mandating a thoracotomy, more liberal use as well as initial attempt of VATS should be advocated as an adjunct for pulmonary TB patients with localized parenchymal lesions.

Differences existed in the grading of image characteristics between patients with medically failed pulmonary TB and MDRTB. Although tuberculoma and aspergilloma posed significant influence on the feasibility of VATS in pulmonary TB with medical failure, the impact was not substantial on MDRTB. As multiple tuberculoma and the existence of aspergilloma indicated a chronically inflamed environment with repeated infection and parenchymal destruction, patients with MDRTB deemed for surgery tended to be devoid of the prolonged disease process. In our institution, patients with MDRTB and cavitation were more likely to undergo surgical intervention than to be medicated alone, suggesting that multiple tuberculoma or aspergilloma were less often encountered and influential than the patients with prolonged medication and disease relapse.

Endobronchial colonization of mycobacterium has been reported as a risk factor for postoperative bronchopleural fistula.^30^ The fear of postoperative bronchopleural fistulas, a devastating complication after a lobectomy or pneumonectomy, hampered early surgical intervention. Although it has been acknowledged that the bronchial stump in patients with MDRTB should be reinforced with a viable intercostal muscle, pleural flaps, or a pericardial fat pad,^1,31^ we did no reinforcement in either group. The bronchial stump healing was associated with peribronchial lymph node or soft tissue coverage.^32^ We believe that avoidance of overt dissection around the bronchus, which is frequently performed in pulmonary diseases of chronic inflammation and adhesion, preserves the healing capability of bronchial stump. Stapled closure of bronchial stump was successful in the VATS group, which could be attributed to the minor extent of inflammation and adhesion. We found the image characteristics in the VATS group were of minor extent in pleural thickening, peribronchial lymph node calcification, and cavitation, which is in concordance with our previous publication.^6^ Notably, aside from the preoperatively positive sputum culture, patients with surgical complications or unfavorable outcome had image characteristics of higher grading. The emergence of medical failure indicated relentless inflammation and parenchymal destruction, so much such that extent of pleural thickening, peribronchial lymph node calcification, cavitation, the amount of operative blood loss, and length of hospital stay were significantly increased in the thoracotomy group. Although patients were expected to remain on anti-TB medications for 24 months after the sputum culture had become negative for TB, early introduction of VATS resection may shorten the whole treatment course.

**FIGURE 1:**
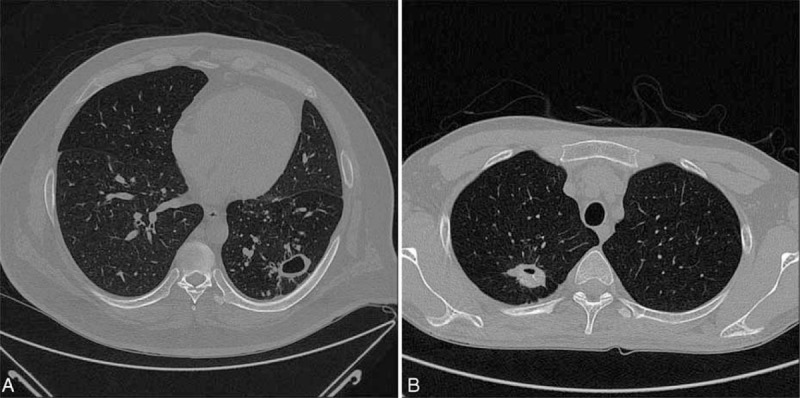
The chest computed tomography scans of our patients with multidrug-resistant pulmonary tuberculosis undergoing video-assisted thoracoscopic surgery wedge resection for (A) left lower lobe and (B) right upper lobe cavities.

We found that pneumonectomies were done only in the thoracotomy group, which had been discussed in our previous report.^7^ The progression of parenchymal destruction in pulmonary TB leads to a gradual shrinking of the thoracic cage with inflammation and adhesion. Unlike a pneumonectomy performed for lung cancer patients, the narrowed intercostal space, shifted mediastinum, severe adhesion in the pleural space, and calcified lymph nodes around the bronchus and vessels in patients with pulmonary TB hinder a VATS pneumonectomy. In fact, patients undergoing pneumonectomy in the thoracotomy group had unfavorable outcomes. Although favorable outcomes could be achieved in patients with medically failed pulmonary TB undergoing pneumonectomy,^31,33^ early intervention before progressive parenchymal destruction with VATS not only preserves pulmonary function but also avoid the deadly complications after pneumonectomy. However, for those with enlarging cavitary lesions as in our patient group, VATS wedge resection could be readily performed before the involvement of the tracheobronchial tree and peribronchial lymph node calcification ensue. Patients treated with VATS wedge resection had favorable outcome without disease relapse (Figure 1). Nonetheless, further patient enrollment and investigation are required before we can reach a solid conclusion.

We also found that when segmentectomy was concomitantly done with lobectomy, 8 of the 11 surgeries were accomplished using thoracotomy. Lesions involving the upper lobe and superior segment of the lower lobe usually had dense adhesion in fissures and VATS vessel control was therefore compromised, making VATS lobectomy with segmentectomy difficult. This operative procedure, however, could still be done using VATS in selected patients. We have used VATS for lingulectomies, posterior segmentectomies of the right upper lobe, and upper division trisegmentectomies of the left upper lobe in patients with open TB and MDRTB. A VATS segmentectomy should hence be taken into consideration as a treatment option to preserve the pulmonary function of patients with pulmonary TB.

Our study does have some limitations because of its retrospective nature and small number of patients. The follow-up period in the VATS group was shorter because of the evolving trend and timing for surgical intervention. Patients with medically failed pulmonary TB deemed for surgery would be timely referred only when VATS therapeutic resection had proved its feasibility and advantage such as less wound pain, shorter hospital stay, and fast recovery for the patients. Even though patients in the VATS group had lesser disease extent and hence lower mycobacterial burden than in the thoracotomy group, it may not be long enough to observe disease relapse in the VATS group. We started using VATS at the beginning of 2007 and it has become the routine procedure for anatomic lung resection since July 2007. Some suitable patients might have been excluded before the learning curve of VATS was overcome. With increased experience in performing VATS, the surgeon's tolerance and perseverance for performing dissection over adhesive and chronically inflammatory areas may gradually increase and thus, more TB patients will benefit from VATS. Consequently, patients with unresolved radiographic lesion despite culture-guided medication with or without disease relapse were also enrolled in addition to those who were sputum smear- or sputum culture-positive at 4 months and those with drug-resistant strain, and contributed to the selection bias.

In conclusion, VATS can be safely performed in meticulously selected patients with medically failed pulmonary TB who demonstrated image characteristics on chest CT scan, such as pleural thickening, peribronchial lymph node calcification, tuberculoma, cavitation, and aspergilloma of minor extent. Before the irreversible parenchymal damage and the resultant adhesion develop, VATS may provide the advantages of minimally invasive surgery and as an effective adjunct to enhance the outcome of medical therapy.
